# Social Support Behaviors and Work Stressors among Nurses: A Comparative Study between Teaching and Non-Teaching Hospitals

**DOI:** 10.3390/bs7010005

**Published:** 2017-01-29

**Authors:** Basil Hameed Amarneh

**Affiliations:** Department of Psychiatric and Community Health Nursing, Faculty of Nursing, Jordan University of Science & Technology, Irbid 22110, Jordan; amarneh@just.edu.jo; Tel.: +962-2720-1000

**Keywords:** work stressors, social support behaviors, Jordan, nurses, hospitals

## Abstract

Purpose: The concept of “work stressors” has been well studied. However, in the field of nursing, studies concerning social support behaviors are limited. The aim of this study was to compare nurse work stressors, social support behaviors, and predictors of these variables among nurses in Jordanian teaching and non-teaching hospitals. Design: A convenience sampling technique and a comparative quantitative research design were used in the current study. Two hundred and ninety-one nurses were recruited from five teaching hospitals, and 172 were recruited from eight non-teaching hospitals in Jordan. Methods: The Nursing Stress Scale (NSS) and the Inventory of Social Supportive Behaviors (ISSB) were used to collect data. Results: The studied variables differed across hospitals. In some subscales, as well as in some individual items of the scales, nurse work stressors and social support behaviors differed between teaching and non-teaching hospitals. In teaching hospitals, the work shift was the only predictor of nurses’ work stressors, whereas the work shift and model of nursing care were predictors of social support behaviors. In non-teaching hospitals, the work shift, level of education, and model of nursing care were predictors of nurse work stressors. Predictors of social support behaviors were marital status, model of nursing, and organizational structure. Conclusions: Regardless of the type of hospital, nurse stressors should be assessed and, once identified, managed by providing various social support behaviors. Clinical relevance: By turning a work environment into a healthy workplace, researchers and nurse leaders believe that improvements can be realized in recruitment and patient safety and quality.

## 1. Introduction

Work stressors vary according to the nature of the workplace. Nurses are at high risk of experiencing work stressors [1,2,3]. These stressors include, but are not limited to, heavy workloads, staff shortages, and restrictive policies [1,2,4,5,6,7]. It is important to identify and use specific measures that are sensitive to work stressors [8]; one such measure is the provision of social support [1,5].

The demands placed on nurses by their job increase their work stressors [7,9] and stressors should be assessed and managed early and in an ongoing manner [6]. Nurses in Jordan experience “workload” as a major stressor in workplaces, and the same is experienced in UK [1,4,5,6]. Dealing with end-of-life issues (death and dying) and conflicts with physicians were the other major stressors in the studied Jordanian hospitals [5]. Regardless of the country, severe stress is serious as it results in burnout, absenteeism, and an increase in turnover [1,5,7,8].

The Jordanian healthcare system is composed of 30 governmental, 60 private, two university-affiliated, and 11 military hospitals. Of these hospitals, seven are accredited teaching hospitals [10]. Teaching hospitals focus on continuing education and training programs more than the other types of hospitals [11]. Because some nurses see policies and standards as extra work stressors, and because policies and standards tend to be more complex and are more rigorously enforced in teaching hospitals, these hospitals may not be regarded as positive work areas [11].

## 2. Purpose and Significance of the Study

There have been many studies concerning factors that are nursing stressors; however, nursing studies regarding social support behaviors have only recently been published [2,5,12]. In general, comparative nursing studies have been done to compare critical care units to non-critical care units [1,9], or in specific clinical settings [13].

In Jordan, there has been a profound change in the health care delivery system by moving towards international accreditation standards. Medical tourism is now a major contributor to the country’s economy. The present study is one of a few that have examined stress at work, as well as social support for nurses in teaching and non-teaching hospitals after these changes [1,5]. The results of this study will help in establishing and designing managerial and leadership interventions for decreasing work stressors, and such interventions include providing social support behaviors. Identifying effective social support behaviors (independent variable) will help in reducing work stressors (dependent variable).

The study aimed at answering the following research questions: (1) What are the differences between teaching hospitals and non-teaching hospitals in relevant, individual nurse variables, related to stressors that nurses in Jordan commonly face in the work place, as well as the social support behaviors that are available for nurses in the work place. (2) What are the predictors of stressors at work in teaching hospitals compared to non-teaching hospitals for Jordanian nurses? (3) What are the predictors of the social support in teaching compared to non-teaching hospitals?

## 3. Background

Worldwide, there are ongoing, frequent, and escalating changes in the healthcare industry. Changes are not just limited to the restructuring of hospitals, and they focus on quality initiatives, changing methods of reimbursement, and the incorporation of advanced technology into clinical settings [3]. Such changes will cause work stressors for many health care professionals; however, nurses are more prone to these stressors, as they have more direct and frequent exposure to clients and their families than other health care professionals do [1,2,3]. The provision of an appropriate social support system of behaviors may help to reduce stressors for nurses at work [1,5].

Work stressors are excessive pressure or demands that induce adverse reactions; they constitute about a third of all new incidents of poor health. Reports indicate that 13.8 million working days were lost to work-related stressors such as depression and anxiety in 2006 and 2007 [14].

Work stressors are wide-ranging; they include, but are not limited to, inadequate social support, dealing with end-of-life issues, increased workload, uncertain treatments, conflicts with MDs, insufficient preparation, problems with peers or supervisors, and handling patients and their significant others [3,8,15,16,17].

Prolonged and severe stress can result in serious physiological and psychological disturbances [2,6,7,8,12]. Decreasing work stressors have been shown to positively influence employees’ commitment to work, job performance and productivity, retention of staff and recruitment, client satisfaction, and the image and reputation of the organization [14].

According to the UK National Work-Stress Network Newsletter, stress is the biggest problem faced in workplaces. The most common triggers for rising stress among employees are reportedly high workloads, job cuts, and rapid organizational changes. In light of these facts, stress management should be a priority in the United Kingdom [4]. However, one researcher reported that, in the United Kingdom, stress prevention activities are generally limited to larger organizations; the researcher also reported the lack of a systemic approach or its infrequent use in the evaluation of the effectiveness of stress interventions [6].

Social support is defined as interrelated social relations and connections that help in the coping and dealing of individuals with stressful life situations [2,12]. Social support lowers the severity of these stress factors [2], buffers the undesirable effects of job demands, and decreases feelings of exhaustion [18].

In their literature review, Michie and Williams concluded that poor social support is one of the key work factors associated with psychological illnesses and absenteeism in staff in the United Kingdom. Poor social support is also a major stressor for nursing students [8].

In another study, researchers reported that heavy workloads, as well as handling end-of-life issues, were common sources of work stress for nurses in Jordan. According to these researchers, receiving emotional support is the social behavior that nurses in Jordan experience as helpful when dealing with stressful events. They reported a significant correlation between nurse work stressors and received social support [5]. Likewise, they found significant correlations between work stressor factors, worked shifts, education level, and modes of nursing care. They also reported higher correlations between social support behaviors, work commitment, and hospital unit/ward decision-making styles. Predictors of work stressors include shifts worked, a nurse’s level of education, and the mode of nursing care, while the predictors of social support behaviors include shifts worked, the nursing care model, marital status, and hospital unit/ward organizational structures [5]. Prior scientific work has demonstrated that nurses working in university hospitals report higher levels of stress than nurses working in government hospitals; in a comparative study between units and wards, it was reported that greater nurse job stress factors and poor social support behaviors were more evident in units than in wards [1]. More specifically, hospital units scored higher than hospital wards on the “conflict with physicians” subscale of the Nursing Stress Scale (NSS). Units also scored higher than wards on the “emotional support” and “tangible assistance” subscales of the Inventory of Social Supportive Behaviors (ISSB). Worked shifts, the mode of nursing care, and the educational level projected nurses’ job stressor factors in hospital units and wards. The mode of nursing care was a mutually estimated predictor for social support behaviors in hospitals unit/wards [1].

Aiming to investigate the predictors of levels of anxiety and depression among nurses, a study of 870 nurses in South England revealed that social support was among the significant factors that negatively affected anxiety and depression (*p* = 0.001) [19].

A published report, which was not limited to Jordanian hospitals, revealed that teaching hospitals, compared to non-teaching hospitals, differ in terms of quality of care, costs, and outcomes, especially in areas related to mortality rates and the addition to the length of the hospital stay, but did not report on the differences in relation to work stressors and social support behaviors [20]. In one research study, it was found that health care professionals in teaching hospitals experienced greater levels of distress and suffered from higher levels of stress at work than did professionals in non-teaching hospitals [21]. Although professionals in non-teaching hospitals experienced less overall job satisfaction, as well as less job autonomy, they showed more satisfaction with work hours than professionals in teaching hospitals. In his thesis, in New York, Carter indicated that nurses showed higher job satisfaction and greater retention rates if they worked in teaching hospitals versus nurses working in non-teaching hospitals [22]. Although the long-term care setting is different than the health care setting, a recent study in West Virginia (USA) on nurses working in long-term care facilities revealed that job demands (greater occupational stress) were associated with more emotional exhaustion, more depersonalization, and less personal accomplishment. Job resources (support from supervisors and friends or family members, reassurance of worth, opportunity for nurturing) were associated with less emotional exhaustion and higher levels of personal accomplishment [23].

## 4. Methods

### 4.1. Ethical Statement

Before data collection, the university ethical committee approved the study protocol, the methodology of research, the anonymity of the participants, and the protection of identity, privacy, and the handling of data. It was explained to participants that completing and submitting the survey automatically meant that they gave consent. The participants did not receive any incentives for participating in the study.

### 4.2. Design, Sample and Settings

This study used a comparative quantitative research design to compare the social support behaviors and the work stress factors of nurses working in teaching hospitals versus non-teaching hospitals, and to identify the predictors of those variables. In 2010, after obtaining permission to conduct the study, participating hospital administrators and nurses were contacted through their nurse managers and were invited to be a part of the study, while being assured that participation was voluntary. Using a convenience sampling technique, 463 nurses were recruited out of 700 possible participants, 291 nurses from five teaching hospitals and 172 from eight non-teaching hospitals; the overall response rate was 66.3%. The study was open to all nurses working in hospital settings. To obtain a large sample, no exclusion criteria were set. The overall results were shared with nursing and hospital administrators.

## 5. Data Collection Procedures

### 5.1. Instruments

The Nursing Stress Scale (NSS) is the best-known and most widely used scale in nursing. It is a 34-item scale, developed to measure the frequency of and the major sources of stress experienced by nurses in hospital units. NSS was used to measure nurse’s work stressors [24]. Seven factors comprise the NSS: the physical environment; four from the psychological environment, and two from the social environment of the hospital.

The second instrument used was the Inventory of Socially Supportive Behaviors (ISSB), which is used to measure social support behaviors [25]. The ISSB has 40 items, with a five-point Likert-type scale. The ISSB has three subscales: the Guidance Scale, the Emotional Support Scale, and the Tangible Assistance Scale. A mean score over three was in favor of social support behavior.

The demographic survey was developed in order to get an array of characteristics: gender, age, marital status, worked shifts, time commitment to work, educational level, experience in nursing, experience in the current area of work (in years), average daily registration, type of hospital unit/ward, hospital ward/unit structure, mode of nursing care, hospital ward/unit’s style in making decisions, and hospital type.

The participants spent 15 min, on average, completing the surveys.

### 5.2. Data Analyses

Descriptive and inferential statistics were estimated using a 0.05 significance level. The Statistical Package for Social Sciences software was used for data analyses [26]. The nonparametric statistics Chi-Square test (χ2) was used to compare variables between hospitals (teaching and non-teaching), and the parametric statistics using one-way ANOVA were used to compare social support and nurse work stressors and between hospitals (teaching vs. non-teaching). Variables that influenced nurse work stressors and social support behaviors were predicted using stepwise multiple regressions [27]. All variables were entered into each stepwise regression model to allow for the assessment of the most influential variables in explaining the variance in the dependent variable [28].

## 6. Results

### 6.1. Instrument Validation

In estimating the Cronbach’s alpha for the Nursing Stress Scale (NSS), the current study indicated that the Cronbach’s alpha value was 0.90, which is very close to the original scale reporting (0.92).

The second instrument used was the Inventory of Socially Supportive Behaviors (ISSB); the original scale showed a higher reliability, as Cronbach’s alphas indicate that it ranges between 0.92 and 0.94, while in the current study they ranged between 0.93 and 0.94.

### 6.2. Comparison of Nurse and Organizational Characteristics

Although there were no significant differences between teaching and non-teaching hospitals with respect to the standard deviations of demographic and organization characteristics, answers to our first research question revealed significant differences in the following variables: *Shift worked*: Almost 47% (*n* = 135) of nurses in teaching hospitals were working 12 h shifts, whereas 48% (*n* = 82) of those in non-teaching hospitals were working rotating shifts (*p* = 0.001); long shifts are more common in teaching hospitals when compared to non-teaching hospitals. *Commitment to work*: 92.3% (*n* = 268) of nurses in teaching hospitals were working full-time versus 85.3% (*n* = 146) in non-teaching hospitals (*p* = 0.017). *Level of education*: 78.1% (*n* = 228) of nurses in teaching hospitals held a baccalaureate versus 60.5% (*n* = 104) in non-teaching hospitals (*p* < 0.001). *Years of experience in nursing*: 72.2% (*n* = 210) of nurses in teaching hospital had less than five years’ tenure compared 53.5% (*n* = 91) in non-teaching hospitals; on the other hand, 27.8% (*n* = 81) of nurses had more than five years’ tenure in teaching hospitals compared to 46.5% (*n* = 79) in non-teaching hospitals (*p* < 0.001). *Years of experience in current area of work*: 31.8% (*n* = 92) of nurses in teaching hospitals had three to four years of such experience, versus 31.4% (5 = 54) with one to two years in non-teaching hospitals (*p* < 0.001). *Patient numbers*: 26.4% (*n* = 76) of nurses in teaching hospitals indicated that their unit/ward’s patient count exceeded 20 patients/day compared to 31.4% (*n* = 54) in non-teaching hospitals (*p* < 0.001). *Organizational structure*: Although the percentages are very low, almost 15% (*n* = 43) of nurses in teaching hospitals indicated that their unit/ward’s organizational structure was unclear versus 9.3% (*n* = 16) in non-teaching hospitals. *Model of nursing*: 42.8% (*n* = 125) of nurses in teaching hospitals reported they used the primary nursing model versus 23.9% (*n* = 41) in non-teaching hospitals (*p* < 0.001) (Table 1).

### 6.3. Work Stressors and Social Support Behaviors: Differences across Hospitals

Significant differences were noted in some nurse work stressors, namely uncertainty concerning treatment (*p* < 0.001), inadequate preparation (*p* < 0.001), and lack of support (*p* = 0.017) (Table 2). Regarding individual questions within NSS, there were some significant differences. With regard to individual questions within the NSS, there were some significant differences in floating to other units that are short staffed (*p* < 0.001); difficulty in working with nurses outside the unit (*p* = 0.005); criticism by supervisors (*p* = 0.012); physicians ordering what appear to be inappropriate treatments for patients (*p* = 0.019); too many non-nursing tasks required (*p* < 0.001); insufficient time to provide emotional support to patients (*p* = 0.011); insufficient time to complete all nursing tasks (*p* = 0.014); not knowing what a patient or patient’s family should be told about the patient’s medical conditions and their treatments (*p* = 0.029); and too few staff to adequately cover the unit (*p* < 0.001) (Table 3A). As regards individual questions in the ISSB, the only significant difference was in response to “when nurses went to someone who could take action” (*p* = 0.022) (Table 3B).

### 6.4. Predictors of Work Stressors and Social Support Behaviors for Nurses (Teaching Hospitals)

In teaching hospitals, the type of work shift was the only predictor of these nurse work stressors (*R*^2^ = 0.018, *F* = 5.16, *p* = 0.024), explaining 13% of the variance, whereas the work shift (*R*^2^ = 0.078, *F* = 24.10, *p* < 0.001) and model of nursing care (*R*^2^ = 0.100, *F* = 15.62, *p* = 0.010) were predictors of social support behaviors. The variance in the shift worked and the model of nursing explained 28% and 3.6%, respectively, indicating the shift worked as the major contributor (Table 4).

### 6.5. Predictors of Work Stressors and Social Support Behaviors for Nurses (Non-Teaching Hospitals)

In non-teaching hospitals, the work shift (*R*^2^ = 0.080, *F* = 14.23, *p* < 0.001), level of education (*R*^2^ = 0.142, *F* = 13.45, *p* = 0.001), and model of nursing care (*R*^2^ = 0.177, *F* = 11.64, *p* = 0.009) were found to predict these nurse work stressors, with a variance explaining 28.3% for the shift worked, which is the highest predictor, 9.4% for the level of education, and 4.4% for the model of care, respectively; the total model explains 4 42.1%. The marital status (*R*^2^ = 0 .057, *F* = 9.87, *p* = 0.002), model of nursing (*R*^2^ = 0.106, *F* = 9.68, *p* = 0.0003), and organizational structure (*R*^2^ = 0.138, *F* = 8.64, *p* = 0.016) predicted social support behaviors with a variance of 23.8% for marital status, 8.8% for model of nursing care, and 4.5% for unit’s/ward’s organizational structure, showing that marital status contributed more to social support (Table 5).

## 7. Discussion

In this study, all the differences in the subscales and the individual items in the stressors scale were higher in teaching hospitals. This indicates that teaching hospitals are associated with greater stressors. High work stressors and low social support behaviors have been reported in both the United Kingdom and Jordan [1,4,5,8].

In this study, nurses in teaching hospitals, compared to non-teaching hospitals, differed in all variables studied, except for gender, marital status, age, and perceptions about the ward’s/unit’s decision-making style. For example, non-teaching hospitals in Jordan include private hospitals, which hire more part-time and diploma nurses, although it is not documented by research, and private hospitals implement this strategy in order to minimize costs. Senior nurses, with many years of experience, are more often employed in non-teaching hospitals, whether they are owned by the government or privately run. In Jordan, nurses who retire from governmental hospitals are usually re-employed at private hospitals under special contracts with higher wages [28]. This trend could also apply to years of experience in a current area of work; this represents an investment in a nurse’s experience [11,28], and experienced nurses often come from different sectors, such as military or teaching hospitals. Non-teaching hospitals in Jordan have higher patient numbers than teaching hospitals, which is due to the nature of health insurance in the country.

One of the surprising results of this study was that, although they accounted for a low percentage in both settings, nurses in teaching hospitals, more than in non-teaching hospitals, reported that the organizational structure of their own hospital was “unclear”.

There is currently a tendency in Jordan towards applying the primary nursing care delivery model. Teaching hospitals are more advanced in this regard because of the particular policies and procedures applied at these hospitals, including those concerning the appropriate nurse-to-patent ratio [11].

Teaching hospitals scored higher than non-teaching hospitals on three subscales of the NSS, namely in lack of certainty concerning treatment, insufficient preparation, and absence of support. The present study is consistent with prior studies [5,17]. At the level of individual items in the NSS, work stressors were higher in teaching hospitals. Although not documented, nurses may not perceive these hospitals as providing “supportive work environments” because they have many policies and procedures [11]; therefore, nurses may opt to work in non-teaching hospitals. Thus, nursing shortages will occur more often in teaching hospitals and such shortages are also associated with increased workloads [28]. “Floating” is a widely used strategy in both developed and developing countries in order to compensate for nursing shortages [29]. Due to the increased workload in teaching hospitals, nurses may not have enough time to provide emotional support to their patients and to complete all nursing tasks [5,17,30]. An increased workload is also associated with nurses performing too many non-nursing tasks in order to compensate for shortages in other health-related and clerical jobs [31].

At teaching hospitals, nursing supervisors tend to direct more criticism against nurses because of the excessive requirements required of them with respect to hospital policies and procedures [11]. Because teaching hospitals impose so many policies on nurses, they may not know what to tell patients’ families, or patients themselves, regarding their medical conditions; in Jordan, only attending physicians are allowed to disclose information to a patient and/or relatives [11]. Other healthcare professionals, patients, and patients’ families can interpret such uncertainties as “a problem in nurses’ knowledge”.

The only significant difference in individual items with the ISSB was “when nurses went to someone who could take action”. This was reported as being advantageous for nurses at teaching hospitals, possibly due to the presence of strong leaders in areas of decision-making at these hospitals [18,32].

The current study shows that nurses are rotated on day and evening shifts, which may explain why the worked shift predicts nurse work stressors in the two types of hospitals (teaching and non-teaching) [5]; nurses in these hospitals are not fixed to a single shift, and the worked shift can interfere with a person’s physical and social well-being. Therefore, more social support behaviors should be provided to nurses who work these shifts, especially if they are married and are, therefore, required to handle additional roles, such as spouse and/or parent [5].

Many nurses in non-teaching hospitals hold baccalaureate degrees; however, these nurses may not receive as much staff development and advancement opportunities as nurses in teaching hospitals. As mentioned earlier, experienced nurses are employed more often in non-teaching hospitals, with no opportunities for further staff development. Assuming that these nurses are better prepared by experience, this could be another work stressor in non-teaching hospitals [5].

Appropriate staffing is critical for good nursing care, as well as for minimizing stressful work. In non-teaching hospitals, the functional model of care is commonly practiced. A major shortcoming of this type of care delivery is the fragmentation of care, which can lead to a patient’s problems being overlooked because they did not fit into a defined assignment, whereas adopting a team model approach, such as with team nursing, which is a system that distributes the care of a patient among a team that works together to provide for said person, requires an acceptable nurse-to-patient ratio, which is another problem due to staff shortages and greater workloads [16,29]. Accordingly, the use of either of these models could be viewed as a work stressor in non-teaching hospitals [5]. On the other hand, the use of the primary nursing care model in teaching hospitals, and team and functional models in non-teaching hospitals, imposes a greater need for nurses to do jobs in fields related to dealing with other teams, themselves, and patients [16,29].

In general, because vertical organizational structures are characterized by centralized decision-making, the provision of additional social support behaviors should be mandatory where this decision-making style is used to minimize its effects [5].

The results of this study have several implications with respect to practice, education, and research. Concerning practice, there is a significant need to increase nursing staff numbers and to relieve nurses of non-nursing workloads [5,31]. Adequate staffing will help nurses to perform their tasks safely with fewer stressors [5], likely resulting in an increase in nurse and patient satisfaction, as well as improved quality of nursing care [11,28].

Concerning education, courses for today’s students and tomorrow’s nurses should emphasize active listening, interpersonal relationships, and communication at both the undergraduate and graduate levels. At-work and in-service training and staff development programs should educate nurses in various areas related to stress sources at work, in addition to social support and acceptable behaviors [5].

With respect to the research, a major limitation of the current study is that it may not be generalizable to the entire population of nurses in Jordan due to the recruiting of a sample without inclusion criteria; thus, future research should study a larger, random sample over wider geographical areas.

For future research, there is a need for a qualitative study of nurses in different settings in order to better understand their needs in terms of better quality of work.

## 8. Summary and Conclusions

The results of some of the studied variables showed a significant level of differences between the two types of hospitals. Teaching hospitals scored higher than non-teaching hospitals in uncertainty concerning treatments, inadequate preparation, and lack of support. The social support behaviors subscale did not show any significant difference between the two types of hospitals. There were some significant differences at the level of individual items in the NSS; work stressors were more frequent at teaching hospitals. At the level of individual items in the ISSB, the only significant difference was when nurses went to someone who could take action; this action was considered to be advantageous for nurses at teaching hospitals. The work shift predicted nurse work stressors in both teaching and non-teaching hospitals.

Nurse work stressors should be assessed and, when identified, managed as early as possible by introducing various managerial and leadership interventions. These interventions can include training in social support behaviors. In addition, decreasing work stressors will promote a higher quality of patient care, as well as a higher quality of life for nurses.

## Figures and Tables

**Table 1:** Characteristics of nurses and hospital organizational factors (*n* = 463).

Variables	Teaching (*n* = 291)		Non-Teaching (*n* = 172)		X^2^ DF	*p*
	*n*	%	*n*	%		
*Shift worked*						
Day (12 h)	135	46.6	16	9.4	91.24, 5 DF	0.001
Evening (12 h)	30	10.3	7	4.2		
Day (8 h)	46	15.8	44	25.7		
Evening (8 h)	9	3.1	11	6.5		
Night (8 h)	2	0.6	11	6.5		
Rotating (A, B, C)	69	23.6	82	47.7		
*Commitment to work*						
Full-time worker	269	92.3	146	85.3	5.87, 1 DF	0.017
Part-time worker	22	7.7	25	14.7		
*Level of education*						
Associate	14	4.8	14	8.1	21.47, 4 DF	0.001
Diploma	38	13.0	48	27.9		
Baccalaureate	227	78.1	104	60.5		
Master	11	3.8	4	2.3		
Doctorate	1	0.3	2	1.2		
*Years of experience as a nurse*						
Less than one year	61	20.9	15	8.8	27.30, 4DF	0.001
1–2 years	60	20.6	44	25.7		
3–4 years	89	30.5	32	18.6		
5–9 years	53	18.3	52	30.2		
10 years or more	28	9.6	27	15.7		
*Years of experience in current setting*						
Less than one year	81	27.7	18	10.7	33.83, 4 DF	0.001
1–2 years	68	23.3	54	31.4		
3–4 years	92	31.8	41	23.8		
5–9 years	34	11.6	35	20.5		
10 years or more	16	5.6	23	13.6		
*Unit/ward’s daily census*						
1–5 patients	71	24.3	24	14.0	23.60, 4 DF	0.001
6–10 patients	63	21.6	20	11.6		
11–15 patients	47	16.1	32	18.6		
16–20 patients	32	11.0	40	23.3		
21 and more patients	77	26.4	54	31.4		
*Unit/ward’s organizational structure*						
Vertical	115	39.5	65	37.8	16.83, 3 DF	0.001
Horizontal	91	31.3	62	36.0		
Matrix	42	14.5	29	16.9		
Unclear structure	43	14.7	16	9.3		
*Model of nursing care*						
Primary	125	42.8	41	23.9	20.90, 3 DF	0.001
Team	96	32.9	72	41.9		
Functional	53	18.4	52	30.2		
Unclear model	17	5.9	7	4.0		

Some numbers do not total to 464 due to missing data; DF, degree of freedom.

**Table 2:** Subscales of nurse work stressors and social support behaviors (*n* = 463).

Subscales	Number of Items	Teaching (*n* = 291)	Non-Teaching (*n* = 172)	F	*p*
		$\bar{X}$ (SD)	$\bar{X}$ (SD)		
*Nurse work stressors*					
Death and dying	7	16.41 (4.06)	16.12 (3.84)	0.569	0.451
Workload	6	11.29 (3.01)	11.06 (2.58)	0.737	0.391
Conflict with physicians	5	6.68 (1.85)	6.65 (1.769)	0.051	0.821
Conflict with other nurses	5	6.54 (2.03)	6.47 (1.84)	0.147	0.701
Uncertainty concerning treatment	5	11.99 (3.28)	10.88 (2.845)	13.502	0.001
Inadequate preparation	3	15.12 (3.56)	13.54 (3.495)	21.585	0.001
Lack of support	3	11.73 (3.04)	11.06 (2.655)	5.765	0.017
*Social support behaviors*					
Guidance	15	37.24 (11.09)	36.49 (9.453)	0.549	0.459
Emotional support	15	37.31 (11.11)	37.34 (9.689)	0.001	0.977
Tangible assistance	10	21.78 (7.38)	20.59 (6.040)	3.206	0.074

**Table 3:** Individual nurse work stressors and social support behaviors (*n* = 463). (**A**) Individual nurse work stressors; (**B**) Individual social support behaviors.

**(A)**				
**Items**	**Teaching (*n* = 291)**	**Non-Teaching (*n* = 172)**	**F**	***p***
	$\bar{X}$ **(SD)**	$\bar{X}$ **(SD)**		
Floating to other units that are short staffed	2.59 (1.00)	2.09 (0.938)	27.761	0.001
Difficulty in working with nurses outside the unit	2.42 (0.903)	2.17 (0.970)	7.866	0.005
Criticism by a supervisor	2.38 (0.920)	2.16 (0.874)	6.399	0.012
A physician ordering what appears to be inappropriate treatment for a patient	2.41 (0.878)	2.22 (0.806)	5.518	0.019
Performing too many non-nursing tasks	2.74 (0.942)	2.24 (0.896)	31.786	0.001
Insufficient time to provide emotional support to a patient	2.58 (1.01)	2.34 (0.981)	6.504	0.011
Insufficient time to complete all of my nursing tasks	2.53 (1.00)	2.30 (0.967)	6.039	0.014
Not knowing what a patient or a patient’s family ought to be told about the patient’s medical condition and its treatment	2.35 (0.837)	2.17 (0.845)	4.819	0.029
Shortage of staff to adequately cover the unit	2.79 (0.998)	2.41 (0.978)	16.573	0.001
**(B)**				
**Items**	**Teaching (*n* = 291)**	**Non-Teaching (*n* = 172)**	**F**	***p***
	$\bar{X}$ **(SD)**	$\bar{X}$ **(SD)**		
Nurses go to someone who can take action	2.46 (1.205)	2.20 (1.112)	5.245	0.022

**Table 4:** Stepwise multiple regression of variables predicting nurse work stressors and social support behaviors in teaching hospitals (*n* = 291).

Variables	R	R^2^	Adjusted R^2^	Regression Coefficients	F	*p*
*Nurse work stressors*						
Shift worked	0.134	0.018	0.014	0.134	5.16	0.024
*Social support behaviors*						
Shift worked	0.280	0.078	0.075	0.280	24.10	0.001
Model of nursing care	0.316	0.100	0.093	0.147	15.62	0.010

**Table 5:** Stepwise multiple regression of variables predicting nurse work stressors and social support behaviors in non-teaching hospitals (*n* = 172).

Variables	R	R^2^	Adjusted R^2^	Regression Coefficients	F	*p*
*Nurse work stressors*						
Shift worked	0.283	0.080	0.074	0.283	14.23	0.001
Level of education	0.377	0.142	0.131	0.250	13.45	0.001
Model of nursing care	0.421	0.177	0.162	0.189	11.64	0.009
*Social support behaviors*						
Marital status	0.238	0.057	0.051	−0.238	9.87	0.002
Model of nursing care	0.326	0.106	0.095	0.224	9.68	0.003
Unit‘s/ward’s organizational structure	0.371	0.138	0.122	−0.179	8.64	0.016
